# Benefits and Pitfalls of Secondary Antibodies: Why Choosing the Right Secondary Is of Primary Importance

**DOI:** 10.1371/journal.pone.0038313

**Published:** 2012-06-01

**Authors:** Colleen F. Manning, Angeliki M. Bundros, James S. Trimmer

**Affiliations:** 1 Department of Neurobiology, Physiology and Behavior, University of California Davis, Davis, California, United States of America; 2 Department of Physiology and Membrane Biology, University of California Davis, Davis, California, United States of America; Louisiana State University, United States of America

## Abstract

Simultaneous labeling of multiple targets in a single sample, or multiplexing, is a powerful approach to directly compare the amount, localization and/or molecular properties of different targets in the same sample. Here we highlight the robust reliability of the simultaneous use of multiple mouse monoclonal antibodies (mAbs) of different immunoglobulin G (IgG) subclasses in a wide variety of multiplexing applications employing anti-mouse IgG subclass-specific secondary antibodies (2°Abs). We also describe the unexpected finding that IgG subclass-specific 2°Abs are superior to general anti-mouse IgG 2°Abs in every tested application in which mouse mAbs were used. This was due to a detection bias of general anti-mouse IgG-specific 2°Abs against mAbs of the most common mouse IgG subclass, IgG1, and to a lesser extent IgG2b mAbs. Thus, when using any of numerous mouse mAbs available through commercial and non-profit sources, for cleaner and more robust results each mAb should be detected with its respective IgG subclass-specific 2°Ab and not a general anti-mouse IgG-specific 2°Ab.

## Introduction

Immunolabeling of target antigens on immunoblots, in tissue sections, in cultured cells, and in preparations bound to multiwell plates, is critical to many areas of basic and clinical research, as well as clinical laboratory science. The utility, quality, and reliability of these diagnostic techniques depend on optimizing every aspect of the procedure, including the characteristics of the sample, the effective application of rigorous techniques of sample preparation, and the labeling procedure itself [1], [2]. It is generally recognized that adhering to the highest standards in the choice of primary antibody (1°Ab) employed in these procedures has a major impact on the quality of immunolabeling, and on the reliability of the information obtained [3]–[6]. In most cases the 1°Ab itself is not labeled, such that detection of the bound 1°Ab requires a labeled secondary Ab (2°Ab). As such, the quality and reliability of the wide variety of commercially available 2°Abs is also important for Ab-based labeling applications. However, for the most part the impact of a 2°Ab choice on an experimental outcome is rarely considered or evaluated to the same extent as the choice of 1°Ab.

Mammalian immune systems make a wide variety of immunoglobulin (Ig) molecules that differ not only in their target specificity, as defined by the hypervariable regions of their heavy and light (H+L) chains, but also by their *in vivo* functionalities as defined by their heavy chain constant regions. Many but not all mammals generate different subclasses of IgG, the predominant Ig class in the adaptive immune response. Humans, mice, and rats have multiple IgG subclasses, whereas rabbits have only a single class of IgG [7]. Broad specificity 2°Abs (*e.g.*, recognizing all IgG H+L chains), as well as those that have been purified to have specificity for a single IgG subclass (*e.g.*, anti-mouse IgG1, IgG2a, or IgG2b) are readily available for the standard host species used for generating 1°Abs. Most laboratories prefer to use general anti-IgG 2°Abs, given their broad utility for detecting any IgG 1°Ab raised in that species.

Simultaneous detection of multiple targets in a single sample reduces many problems associated with sample heterogeneity. This is particularly relevant in immunohistochemistry, where labeling in adjacent sections is an imprecise way to demonstrate antigen colocalization. Valid colocalization of multiple targets in a single sample by light microscopy typically requires simultaneous multiplex immunofluorescence labeling with 1°Abs specific for the individual targets. The most common application of this technique is to apply 1°Abs raised in different species, followed by species-specific anti-IgG 2°Abs labeled with different fluorescent dyes. This approach, however, requires the availability of validated 1°Abs raised in distinct species. As the most commonly available 1°Abs are raised in rabbits (for polyclonal Abs or pAbs) and mice (for mAbs), simultaneous multiplex labeling using an approach employing Abs raised in different species is often restricted to two targets. While there exist more complicated serial and/or amplification labeling steps that allow for the sequential use of two or more 1°Abs from the same species [8], [9], the application of these approaches has been limited by their complexity and length, and the extreme care that must be taken to avoid cross-labeling of different 1°Abs with the same 2°Ab. All mouse IgG mAbs exist as a single IgG subclass (typically IgG1, IgG2a or IgG2b). The ability to reliably detect mouse mAbs of different IgG subclasses adds great utility to multiplexing applications, given the enhanced flexibility of combining mouse mAbs of different IgG subclasses from the large catalog of mouse mAbs in current use in basic and clinical diagnostic applications.

Here we demonstrate the advantages of using anti-mouse IgG subclass-specific (SCS) 2°Abs for robust and reliable multiplex labeling of target proteins in a variety of applications, including immunoblots, immunohisto- and immunocyto-chemistry, and microplate binding assays. We also present unexpected results demonstrating that general anti-mouse IgG H+L (HL) 2°Abs display a prominent detection bias against mAbs of the IgG1 subclass and that this bias compromises mouse mAb labeling in multiple procedures. That this bias exists, and can be simply overcome by using SCS 2°Abs, is an important finding that should have a broad impact in enhancing the usefulness of the large catalog of available mouse mAbs, and those being generated in large-scale government-funded efforts that have recently been initiated in the US (*e.g.*, Protein Capture Resource, NeuroMab), Europe (*e.g.*, Affinomics) and elsewhere (*e.g.*, Renewable Protein Binder Working Group [10]). Note that for simplicity we will hereafter use the term “mAb” to refer to those made in mouse, unless specifically stated otherwise.

## Results

### SCS 2°Abs allow for robust and reliable multiplex immunofluorescence labeling of immunoblots

**Figure 1:**
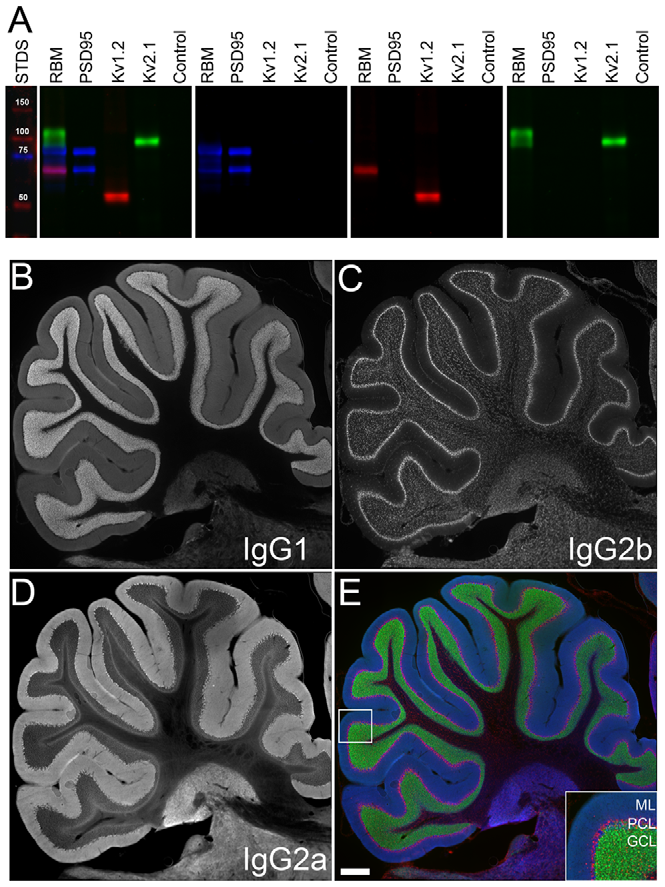
SCS 2°Abs yield robust and reliable simultaneous triple labeling with three mAbs on immunoblots and in rat brain sections. (A) A single immunoblot containing samples of crude rat brain membranes (RBM, 50 µg protein) and extracts of transfected COS-1 cells expressing individual target proteins, or from control cells transfected with an empty plasmid, probed with anti-PSD-95 (IgG2a, blue), anti-Kv1.2 (IgG2b, red) and anti-Kv2.1 (IgG1, green), and SCS 2°Abs. Multicolor panel is original immunoblot; single color panels are images of separated colors. Lane to left shows molecular weight standards in kDa. Note differential post-translational modification of target proteins in brain versus heterologous cells alters their relative electrophoretic mobility. B–E. Images show specific and non-overlapping labeling for (B) Kv4.2 (green), (C) QKI (red), (D) and BK channels (blue), and (E) merge of all three, in a rat brain section, showing the region containing the entire cerebellum. Inset in E shows boxed area of cerebellar cortex. Labels mark the molecular layer (ML), Purkinje cell layer (PCL), and granule cell layer (GCL). Scale bar on Panel E = 500 µm.

Visualization of multiple targets on a single immunoblot is important for comparing the expression levels and molecular characteristics of target proteins, while avoiding problems associated with the heterogeneity inherent in comparing separate immunoblots, and the damage to the sample that can result from serially stripping and reprobing single immunoblots. Simultaneous multiplex labeling of immunoblots is generally performed using 1°Abs generated in different species and species-specific 2°Abs. We tested whether simultaneous multiplex labeling of different targets on immunoblots could be reliably obtained by employing multiple mAbs and SCS 2°Abs. We tested the specificity and reliability of labeling of three different mAbs of different IgG subclasses, recognizing three different brain proteins against a single immunoblot containing samples of a crude rat brain membrane preparation (RBM), and samples prepared from heterologous cells singly expressing each recombinant cognate antigen, or cells transfected with empty plasmid. As shown in Figure 1A, robust specific labeling for each of the individual target proteins was obtained, with little or no detectable crosstalk between signals. The leftmost immunoblot panel shows the single immunoblot imaged to reveal simultaneous labeling of all three mAbs as specifically detected with the three corresponding fluorescently labeled SCS 2°Abs. The subsequent panels to the right show images corresponding to the individual fluorescence channels. In each case the mAbs label the distinct bands in RBM, and a band in heterologous cells expressing the cognate recombinant target, but do not exhibit a detectable signal in heterologous cells expressing alternate targets or transfected with empty expression plasmid. The anti-PSD-95 mouse mAb (IgG2a mAb K28/43 in blue) labels the distinct bands of PSD-95 in RBM, and a band in heterologous cells expressing recombinant PSD-95, but does not exhibit a detectable signal in heterologous cells expressing the Kv1.2 or Kv2.1 voltage-gated potassium channels. Similarly, a mouse mAb against Kv1.2 (IgG2b mAb K14/16 in red) shows robust labeling of Kv1.2 in RBM, and in the sample from heterologous cells expressing Kv1.2, but exhibits no signal in samples from heterologous cells expressing the PSD-95 or Kv2.1. Note that the Kv1.2 pool in brain has a distinct electrophoretic mobility on SDS gels due to N-linked glycosylation [11] and phosphorylation [12]. Similarly, an anti-Kv2.1 mouse mAb (IgG1 mAb K89/34 in green) only labels the Kv2.1 pool in RBM, and the sample from heterologous cells expressing recombinant Kv2.1. Note that Kv2.1 has a microheterogeneous electrophoretic mobility on SDS gels due to extensive multisite phosphorylation [13], the pattern of which is distinct from that on Kv2.1 in the heterologous cell sample [14]. No signal was detected for any of the mouse mAb 1°Abs or 2°Abs in the sample prepared from cells transfected with empty expression plasmid. These results demonstrate the effectiveness and specificity of multiplex labeling of immunoblots using simultaneous application of three different mAbs and their subsequent detection using SCS 2°Abs.

### SCS 2°Abs enable robust and reliable multiplex immunofluorescence labeling of rat and mouse tissue sections using multiple mAbs

We next tested the utility and reliability of mAbs for simultaneous multiplex immunofluorescence labeling of rat brain tissue. As shown in Figure 1B–E, distinct non-overlapping patterns of labeling were obtained when we performed simultaneous triple labeling with three mAbs and SCS 2°Abs in rat cerebellum, in this case for the Kv4.2 voltage-gated potassium channel (IgG1, panel B, green in panel E) in the granule cell layer (GCL), the Quaking RNA binding protein QKI (IgG2b, panel C, red in panel E) in oligodendrocytes throughout the Purkinje cell layer (PCL) and cerebellar white matter, and the large-conductance calcium and voltage-sensitive potassium or BK channel (IgG2a, panel D, blue in panel E) in basket cell terminals and in Purkinje cells located in the PCL, as well as diffuse labeling in the molecular layer (ML). These patterns are consistent with previously published reports of the localization of these proteins in cerebellum [15]–[18].

**Figure 2:**
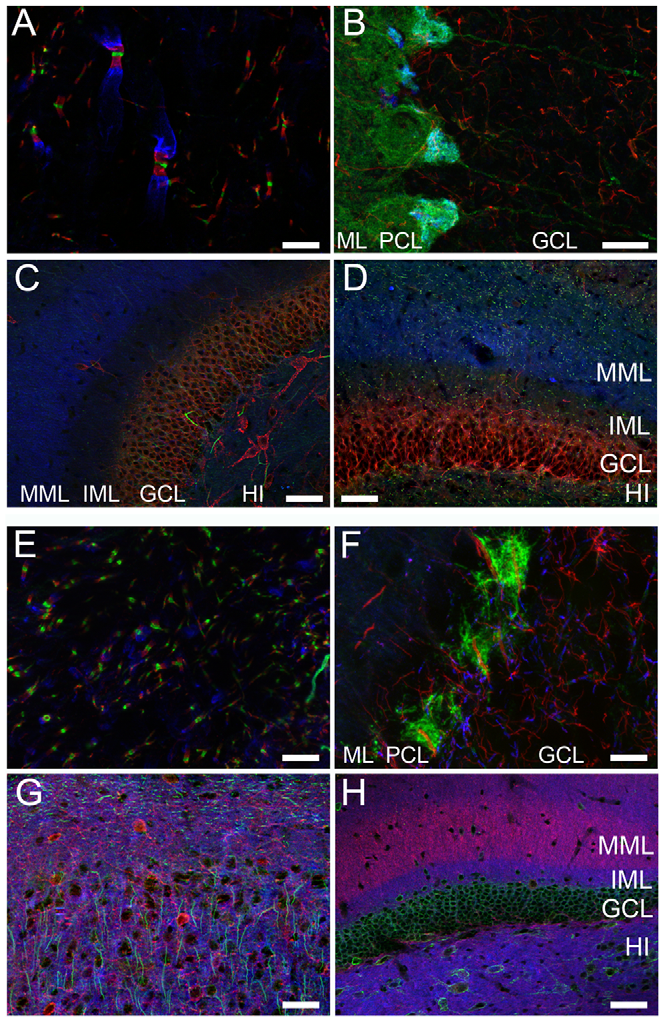
Simultaneous triple labeling with different combinations of three mAbs in rat and mouse brain. Sections from rat (A–D) and mouse (E–H) were simultaneously labeled with three different mAbs, and SCS 2°Abs. (A) Ankyrin-G (green), Caspr/Paranodin (red), and Kv1.2 (blue), in a region of rat hindbrain white matter with myelinated axons containing nodes of Ranvier. (B) BK channel (green), GFAP (red), and Kv1.2 (blue), in a region of rat cerebellar cortex. Labels mark the molecular layer (ML), Purkinje cell layer (PCL), and granule cell layer (GCL). (C) Ankyrin-G (green), Kv2.1 (red), and Kv1.2 (blue), in rat hippocampal dentate gyrus. Labels mark the middle molecular layer (MML), inner molecular layer (IML), granule cell layer (GCL), and hilus (HI). (D) Caspr/Paranodin (green), Ankyrin-G (red), and Kv1.2 (blue), in rat hippocampal dentate gyrus, labels are as in Panel C. (E) mAbs and 2°Abs as in A, but in mouse hindbrain white matter with myelinated axons containing nodes of Ranvier. (F) Kv1.2 (green), Ankyrin-G (red), and GFAP (blue), in a region of rat cerebellar cortex. Labels as in B. (G) Ankyrin-G (green), KChIP1 (red), and Kv1.2 (blue), in mouse cerebral cortex. (H) Kv2.1 (green), Kv1.2 (red), and PSD-95 (blue), in mouse hippocampal dentate gyrus. Labels as in D. Scale bars: A, E, F = 10 µm; B = 20 µm; C, D, G, H = 50 µm.

We next determined whether such reliable simultaneous multiplex labeling using multiple mAbs could be generalized to a variety of mAbs against different targets, and across different rat brain regions, cell types and subcellular domains. In Figure 2A, distinct and non-overlapping labeling of three axonal proteins associated with distinct domains at the node of Ranvier in hindbrain white matter was obtained: Ankyrin-G (green) in the node of Ranvier itself, Caspr/Paranodin (red) in the paranodal domain, and Kv1.2 (blue) in the juxtaparanode. In cerebellum (Figure 2B) specific labeling of the BK channel (green) in the somata, dendrites and axons of Purkinje neurons, and in basket cell terminal pinceau, the glial fibrillary acidic protein or GFAP (red) in glial cell processes throughout the granule cell layer, and Kv1.2 (blue) in basket cell terminal pinceau was observed. In the hippocampal dentate gyrus (Figure 2C), labeling for Kv2.1 (red) localizes in the cell bodies and proximal dendrites of dentate granule cells and interneurons scattered throughout the molecular layer and hilus, for Ankyrin-G (green) to the axon initial segments of these cells, and scattered nodes of Ranvier, and for Kv1.2 (blue) to medial perforant path axons and nerve terminals in the middle molecular layer (MML) of the dentate gyrus, and in scattered juxtaparanodes. Similarly, reliable and specific labeling of Ankyrin-G (red) in axon initial segments of dentate granule cells and nodes of Ranvier, Caspr/Paranodin (green) in paranodal domains of nodes of Ranvier, and Kv1.2 (blue) in medial perforant path axons and nerve terminals in the MML was observed in dentate gyrus (Figure 2D).

We also performed similar experiments on mouse brain sections. Figure 2E demonstrates the reliable reproduction of the labeling seen in rat brain in Figure 2A. Labeling of mouse cerebellum (Figure 2F) yielded specific non-overlapping labeling of Kv1.2 (green) in basket cell terminal pinceau, Ankyrin-G (red) in axon initial segments of Purkinje cells and cerebellar granule cells throughout the GCL, and GFAP (blue) in glial cell processes. Specific non-overlapping labeling in mouse cerebral cortex (Figure 2G) was observed for the KChIP1 Kv channel auxiliary subunit (red) in the cell bodies and dendrites of cortical interneurons, Ankyrin-G (green) in the axon initial segments of cortical neurons, and scattered nodes of Ranvier, and Kv1.2 (blue) in nerve terminals diffusely labeled throughout the cortex, and in scattered juxtaparanodes. Reliable and specific labeling was also obtained in mouse dentate gyrus (Figure 2H) using mAbs against Kv2.1 (green) in the cell bodies and proximal dendrites of dentate granule cells and interneurons scattered throughout the molecular layer and hilus, Kv1.2 (red) in medial perforant path axons and nerve terminals in the MML of the dentate gyrus, and in scattered juxtaparanodes, and PSD95 (blue), with labeling in dendrites in the inner molecular layer of the dentate gyrus and in the hilus. These results provide compelling examples of the reliability and specificity obtained when using mAbs of distinct IgG subclasses in conjunction with SCS 2°Abs for simultaneous multiplex labeling of different target proteins expressed in different cell types, subcellular domains, and regions of rat and mouse brain tissue.

### HL 2°Abs exhibit subclass-specific detection bias across multiple 1° and 2°Ab concentrations in a variety of applications

**Figure 3:**
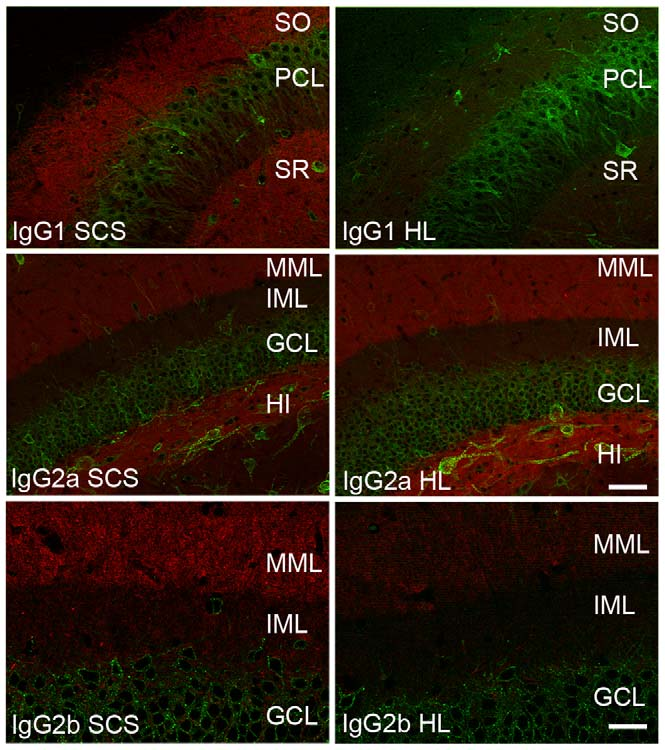
HL 2°Abs show a bias for immunohistochemistry labeling with mAbs of different IgG subclasses. Rat brain sections were labeled with the same concentrations of a single mAb, and a rabbit anti-Kv2.1 pAb, followed by detection with SCS (left column) or HL (right column) 2°Abs, (red), and anti-rabbit IgG (green), each at 1 µg/ml. Top row: anti-Kv4.2 IgG1; middle row: anti-BK channel IgG2a; and bottom row: anti-Kv1.2 IgG2b. Each row was imaged at the same exposure times. Scale bar = 50 µm for panels in top two rows, and 25 µm for panels in bottom row.

Single label immunofluorescence detection of target proteins with mouse IgG mAbs is typically performed using 2°Abs specific for constant regions of HL chains, for example fluorescently-labeled HL. To compare SCS 2°Abs to anti-HL 2°Abs for immunodetection, we labeled rat brain sections with a single mAb (in red), representing one of the predominant IgG subclasses: IgG1, IgG2a, or IgG2b, together with a rabbit polyclonal Ab (in green) as a control for section quality, Ab penetration, and imaging consistency. As shown in Figure 3, the sections labeled with the mAbs and the SCS 2°Abs (left column) yielded more robust, reliable and specific labeling for the IgG1 and IgG2b mAbs than those labeled with the HL 2°Abs at the same concentration (right column). Similar results, with the HL 2°Ab exhibiting a strong detection bias against IgG1 mAbs, and to a lesser extent IgG2b mAbs, were observed for numerous mAbs on rat brain sections (data not shown).

**Figure 4:**
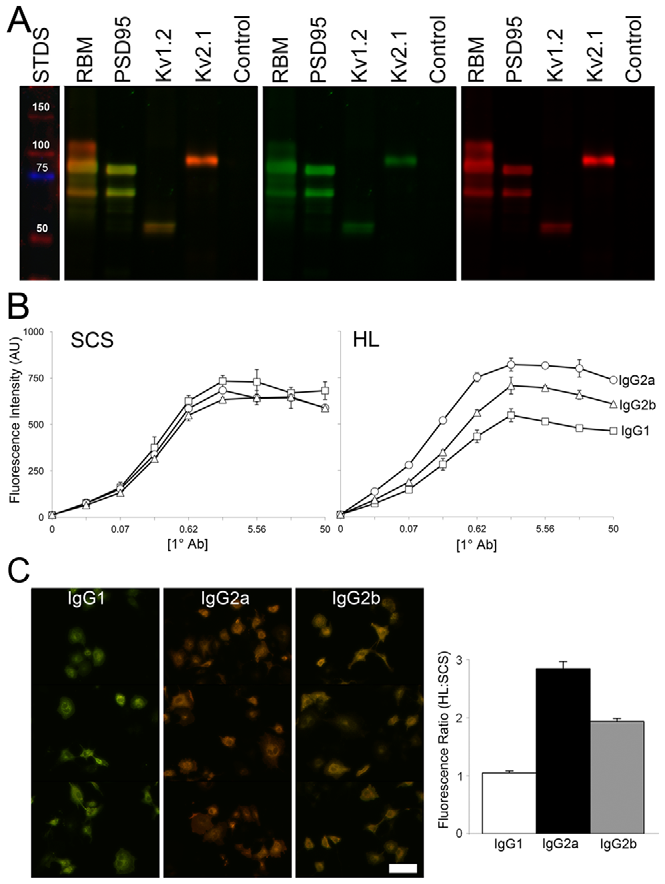
HL 2°Abs show a bias for mAbs of different IgG subclasses in a variety of applications. (A) A single immunoblot containing samples of crude rat brain membranes (RBM, 50 µg protein) and extracts of transfected COS-1 cells expressing individual target proteins, or from control cells transfected with an empty plasmid as labeled, probed with anti-PSD-95 (IgG2a), anti-Kv1.2 (IgG2b) and anti-Kv2.1 (IgG1) mAbs, and HL 2°Ab (green), and a cocktail (1∶1∶1) of SCS anti-IgG1, -IgG2a and -IgG2b 2°Abs (red). Multicolor panel is original immunoblot; single color panels are images of separated colors. Changes in tint reflect bias of HL for (more green) IgG2a>IgG2b>IgG1 (more red). Lane to left shows molecular weight standards in kDa. (B) FLISAs show that IgG subclass bias of HL is present at all concentrations of 1°Abs. Left panel: SCS 2°Abs (each at 1 µg/ml). Right panel: HL 2°Ab. Circles: L76/36 IgG2a; triangles; K14/16 IgG2b; squares: K14/39 IgG1. (C) IgG subclass bias is also present in immunofluorescence labeling of Kv1.2-expressing COS-1 cells. Cells were labeled with mAb as noted, and HL 2°Ab (red), and SCS 2°Abs (green) as detailed in Methods. Changes in red∶green tint reflect bias of HL for (more red) IgG2a>IgG2b>IgG1 (more green). Scale bar = 100 µm. Panel to right is quantitation of immunocytochemistry results from three fields each of three independent samples.

We next tested whether the subclass-specific detection bias observed for HL 2°Abs in immunofluorescence labeling of brain sections was also apparent on immunoblots. Using the same 1°Abs and samples used in Figure 1A, we simultaneously tested HL (green) and a cocktail (1∶1∶1) of SCS (red) 2°Abs for relative immunoreactivity against IgG1, IgG2a and IgG2b mAbs. As shown in Figure 4A, the general HL 2°Ab (in green) shows higher reactivity against the IgG2a anti-PSD95 mAb, as shown by its overall green tint, than for the IgG2b anti-Kv1.2 mAb (yellow tint) and especially for the IgG1 anti-Kv2.1 mAb (red tint). Note that single labeling experiments verified that the presence of the different 2°Abs did not interfere with one another's binding (data not shown). Overall, these immunoblot results reveal a similar profile of detection bias of the HL 2°Ab for IgG2a>IgG2b>IgG1 mAbs as seen with the immunohistochemistry above.

To better quantify these differences and determine their dependence on 1° Ab concentration, we next performed fluorescence-linked immunosorbent assays (FLISAs) using mAbs of different subclasses raised against the same target protein. As shown in Figure 4B (left panel), these mAbs yield similar levels of dose-dependent binding, as detected with their respective SCS 2°Abs. However, when their binding was detected using HL 2°Ab (Figure 4B right panel), we observed the same subclass-specific detection bias with the HL 2°Ab for IgG2a (circles) over IgG2b (triangles) and especially over IgG1 (squares) across all 1° Ab concentrations tested.

To determine whether this IgG subclass bias was also present in immunocytochemistry experiments, we expressed Kv1.2 in transiently transfected COS-1 cells and labeled with the same set of anti-Kv1.2 mAbs, switching the 2°Ab preparations such that now the HL was conjugated to Alexa 594 (red), while the SCS 2°Abs were conjugated to Alexa 488 (green). As shown in Figure 4C, the same detection bias was observed, in that representative images of the Kv1.2-transfected cells labeled with the IgG1 mAb exhibit a green tint, the IgG2b mAb a yellow tint, and the IgG2a mAb a red tint. The graph of the ratio of the anti-HL 2°Ab signal relative to the SCS 2°Ab signal is shown in the right hand panel of Figure 4C, which shows that anti-HL 2°Ab exhibits an ≈3-fold detection bias towards IgG2a relative to IgG1, with IgG2b intermediate (≈2-fold versus IgG1).

**Figure 5:**
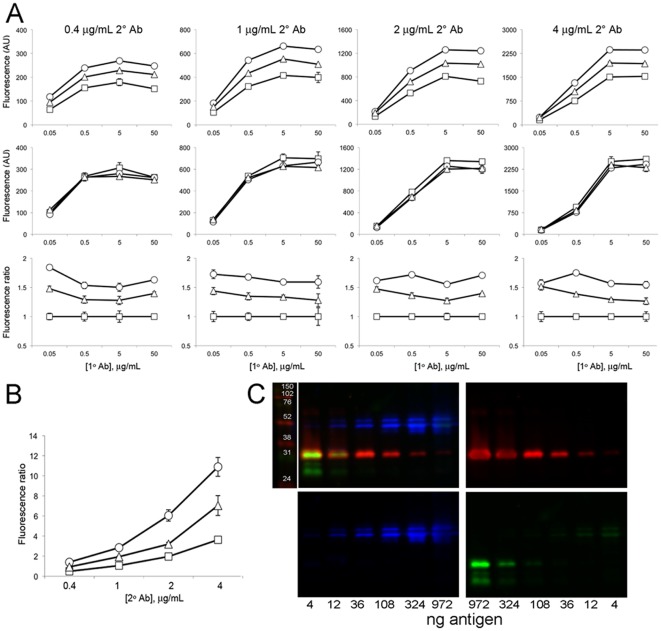
HL 2°Abs exhibit background and detection bias independent of 1° and 2°Ab concentrations. (A) FLISAs showing detection of different concentrations of IgG1 (K14/39, squares), IgG2a (L76/36, circles), and IgG2b (K14/16, triangles) mAbs as indicated by the values on the X-axes, with HL 2°Ab (top row), and respective SCS 2°Abs (middle row), at the concentrations indicated above the columns. Bottom row shows data from the graphs in the top row normalized to values for the IgG1 mAb. (B) HL bias is seen at all 2°Ab concentrations tested in transiently transfected COS-1 cells. Immunofluorescence labeling of Kv1.2-expressing COS-1 cells, probed with 5 µg/mL of IgG1 (K14/39, squares), IgG2a (L76/36, circles), and IgG2b (K14/16, triangles) mAbs and different amounts of HL 2°Ab (red), and the respective SCS 2°Abs (green), as indicated on the X-axis. The Y-axis is the red∶green (HL∶SCS) fluorescence ratio (in arbitrary units). (C) Immunoblots showing lack of crossreactivity in SCS 2°Ab detection of antigens loaded at great excess. Recombinant GST fusion proteins containing different amounts of Kv1.2 and PSD95 antigens, and GST alone, were size fractionated on a single SDS gel and transferred to an immunoblot. Amounts loaded of GST-PSD95 ranged from 4–972 ng, as indicated below lower left panel, and for GST-Kv1.2 and GST alone from 972–4 ng, as indicated below lower right panel. The immunoblot was simultaneously probed with anti-Kv1.2 K14/16 (IgG2b, red), anti-PSD95 K28/43 (IgG2a, blue) and anti-GST N100/13 (IgG1, green), and corresponding SCS 2°Abs. Lane to left of top left panel shows molecular weight standards in kDa. Image reveals a lack of crossreactivity between SCS 2°Abs and bound 1°Abs even under conditions of excess antigen.

We next determined in more detail the dependence of the immunoreactivity on both 1° and 2°Ab concentrations. To better quantify these differences in 2°Ab binding to mouse mAbs of different IgG subclasses, quantitative FLISAs were performed. We analyzed binding to plates coated with a Kv1.2 GST fusion protein (GST-RAK) that contains the binding sites for the three anti-Kv1.2 mAbs detailed above. In Figure 5A, each individual graph represents average fluorescence intensity values for a single 2°Ab concentration, as noted above, and four different 1°Ab concentrations, as given on X-axes. As shown in the top row of Figure 5A, the IgG2a (circles)>IgG2b (triangles)>IgG1 (squares) detection bias exhibited by the anti-mouse IgG H+L 2°Ab is observed across all 1°Ab concentrations tested, and at every concentration of 2°Ab tested. Again, while the HL 2°Ab displayed a bias towards IgG2a and away from IgG1 at all 1° and 2°Ab concentrations tested, the SCS 2°Abs were reliable and equal in reactivity at all 1° and 2°Ab concentrations (Figure 5A middle row). The bottom row of Figure 5A shows the data from the top row normalized to the IgG1 values, to highlight the bias present across all fluorescence intensities.

We next tested whether an increase in 2°Ab concentration could negate the subclass bias seen in immunocytochemistry experiments as in Figure 4C. Cells transiently transfected with Kv1.2 were stained with three anti-Kv1.2 mAb 1° Abs of different IgG subclasses. Each 1°Ab was used at the same saturating concentration, with a cocktail of both SCS and HL 2°Abs applied at four different concentrations. The graph in Figure 5B shows that as we increase the concentration of 2°Ab, the HL subclass bias increases, with the same overall trend as shown in Figure 4B above for the FLISAs.

To establish that the specificity of SCS 2°Abs in multiplexing procedures was present even when the target antigens were present at drastically different expression levels, we analyzed their specificity at detecting three different purified GST fusion protein antigens present on the same immunoblot at a variety of different protein concentrations, up to and including a 243-fold difference in concentration. We used three 1°Abs representing the three major mouse IgG subclasses, anti-Kv1.2 (K14/16 IgG2b in red), anti-PSD95 (K28/43 IgG2a in blue) and anti-GST (N100/13 IgG1 in green). Detection of the anti-Kv1.2 with the red SCS anti-IgG2b 2°Ab is specific to the IgG2b mAb bound to the 32 kDa GST-Kv1.2 protein, with no detectable signal against the IgG2a and IgG1 mAbs bound to GST-PSD95 and GST alone, respectively, even when they are present at a 243-fold excess. Similarly, the blue SCS anti-IgG2a 2°Ab detects the IgG2a mAb bound to 50 kDa GST-PSD95 protein, and does not bind detectably to 1°Abs of other subclasses bound to excess levels of GST-Kv1.2 or GST. The green SCS anti-IgG1 2°Ab detects the anti-GST IgG1 Ab bound to all three GST proteins. Note that the GST-PSD95 sample has additional bands, ranging from 40–55 kDa, representing proteolytic fragments that presumably retain the K28/43 epitope. The GST-Kv1.2 sample has an additional 29 kDa fragment that contains the anti-GST but not the anti-Kv1.2 epitope. Despite the large differences in protein expression levels, as is seen in the various concentrations of GST fusion proteins, and the endogenous proteins in rat brain, the SCS 2°Abs remain specific to their target subclass. Taken together, these experiments show that the differences between the HL and SCS 2°Abs are present at all 1° or 2°Ab concentrations tested in immunohistochemistry, immunoblotting, immunocytochemistry and FLISAs.

### Subclass-specific detection bias is seen in multiple commercial 2°Ab preparations

**Figure 6:**
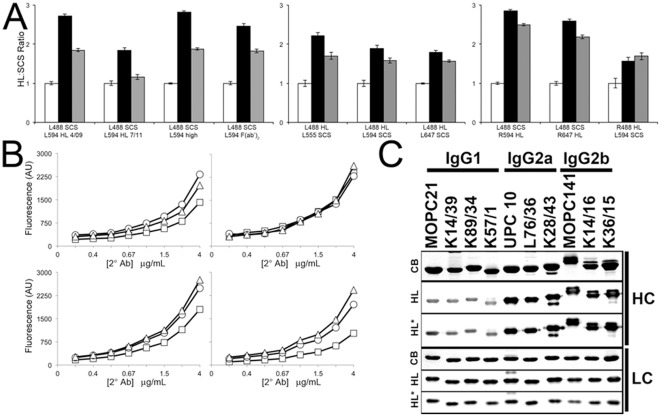
HL detection bias is seen in 2°Ab preparations from different suppliers, with different fluorophores, and with enzyme conjugates. (A) Kv1.2-transfected COS-1 cells were labeled with 1°Ab as in Figure 4C, and HL 2°Ab and the respective SCS 2°Abs, and the ratios of fluorescence intensities from three fields each of three independent samples normalized to the HL/IgG1 ratio. Letters are supplier (L = Life Technologies, R = Rockland), numbers are Alex or DyLight fluorophore conjugates; high: highly adsorbed; fab: F(ab′)_2_ fragment of HL (*e.g.*, L488 SCS is Life Technologies Alexa 488 conjugated SCS). 4/09 and 7/11 refer to two lots of Life Technologies HL. (B) FLISAs showing detection bias of 2°Abs is present at all 2°Ab concentrations. Upper left: Life Technologies HL. Upper right: Life Technologies SCS. Lower left: Jackson ImmunoResearch HL. Lower right: Jackson ImmunoResearch HL (highly cross-adsorbed). (C) HRP conjugated HL secondaries show detection bias by immunoblot. Purified mAb IgG preparations were analyzed by reducing SDS-PAGE and coomassie blue staining (CB), or immunoblotting and detection with two different HRP-conjugated H+L 2°Abs and ECL. HL: Kirkegaard & Perry Laboratories. HL*: Antibodies Incorporated. Note subclass-specific differences in detection of heavy chain (HC) but not light chain (LC) bands in IgG preparations.

To determine whether the results obtained with this initial set of 2°Abs could be generalized across different lots, preparations, fluorophores/conjugates and suppliers, we performed side-by-side comparisons using the immunocytochemistry assays as used in Figure 4B. As shown in the left panel of Figure 6A, two different lots of HL obtained from the same supplier two years apart exhibit comparable subclass bias, as do two different 2°Ab preparations from the same supplier, one highly adsorbed to eliminate species cross-reactivity, and the other a F(ab′)_2_ preparation. We also found that the nature of the fluorophore did not impact the results (middle panel). Finally, we found that HL 2°Abs obtained from different suppliers exhibited the same detection bias (right panel). In Figure 6B, we show that the subclass-specific detection bias of the Life Technologies HL relative to SCS 2°Abs is seen at all 2°Ab concentrations tested (upper panels), and is also seen in two different preparations from yet a third supplier, one a standard HL (lower left panel), the other a highly adsorbed preparation (lower right panel).

We also tested whether this subclass-specific detection bias was observed in horseradish peroxidase (HRP)-conjugated 2°Abs. As shown in Fig. 6C, a comparison of purified mAb IgGs by SDS-PAGE and coomassie blue (CB) staining, and by immunoblotting with HL 2°Abs from two different suppliers, reveals a detection bias against the heavy IgG chains (IgG2a>IgG2b>IgG1) remarkably similar to that seen for the fluorescent 2°Abs. Note that unlike that of the heavy chains, immunoreactivity against the kappa light chains is consistent. In total we used 5 different assays to analyze 27 different 2°Ab preparations, encompassing 12 different SCS and 15 different HL 2°Ab preparations obtained from 5 different companies, and found each of the HL 2°Ab preparations exhibited subclass-specific bias across all concentrations and assays.

### Analysis of knockout mouse brains reveals striking differences in 2°Ab performance

**Figure 7:**
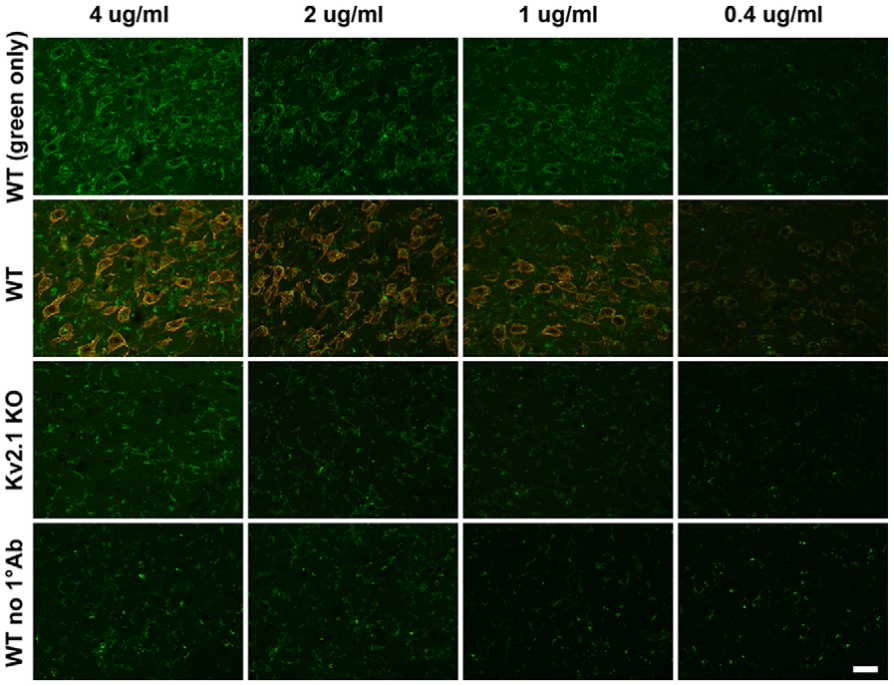
Analysis of knockout mouse tissue reveals increased background of HL 2°Abs. Sections of brains from WT and Kv2.1 knockout (KO) mice were labeled with an anti-Kv2.1 IgG1 mAb, or in vehicle alone (bottom row, no 1°Ab), followed by simultaneous detection with both HL (green) and IgG1-specific (red) 2°Abs. Columns represent samples with different [2°Ab] as in column header. All samples were imaged using identical exposure times. Note that the panels in the top row are the same as those in the WT row but showing the green channel only. Scale bar = 25 µm.

Labeling of samples from knockout mice has become a standard for validating antibody specificity in native tissue [4]. As shown in Figure 7, we labeled brain sections prepared from wild-type (WT) mice, and from mice lacking expression of the Kv2.1 potassium channel (KO), with an IgG1 anti-Kv2.1 mAb, and found robust and specific labeling in WT mice when detected by the SCS 2°Ab (red), and weaker signal and higher background when detected by the HL 2°Ab (green), as shown in the KO sample with 1°Ab, and the WT and KO samples lacking 1°Ab. Diluting these 2°Abs led to a loss of signal, as well as the background labeling observed for the anti-HL 2°Ab. These data reveal that not only do SCS 2°Abs exhibit more robust and reliable detection of bound mAbs in a variety of applications, they also facilitate specific labeling of mouse tissues by mAbs in immunohistochemistry procedures.

## Discussion

One advantage of immunofluorescence-based techniques is the potential for the simultaneous labeling of multiple target proteins in the same sample. This multiplex labeling reduces effort and provides more efficient use of valuable samples. Moreover, it eliminates many of the scientific caveats associated with single probe labeling of replicate samples, or with serial multiplex labeling by stripping and reprobing the same sample. The availability of fluorescent dyes with different excitation and emission characteristics, and high quality filters that allow for acquisition of non-overlapping fluorescent signals from such dyes, allows for simultaneous detection of multiple fluorescent probes in the same sample. The most common Ab-based multiplex labeling procedure involves simultaneously applying multiple 1°Abs, and then using 2°Abs conjugated to different fluorescent dyes to distinguish the sites of 1°Ab labeling. Employing 1°Abs generated in different species has long been recognized as a reliable and effective approach for multiplex immunofluorescence labeling, the availability of a wide variety of mouse IgG mAbs, each of which has a single and defined IgG subclass, offers a similar yet largely untapped potential for multiplex labeling. Here we demonstrated the effectiveness of coupling mAbs with SCS 2°Abs for simultaneous multiplex labeling, and found that this approach yielded robust, specific and reliable signals in a variety of commonly used applications. These studies highlight the utility of employing multiple mAbs of different IgG subclasses in multiplexing experiments, either in combinations with one another, or in conjunction with antibodies raised in other species (*e.g.*, rabbits, guinea pigs, etc.), and further highlight the advantages of using SCS 2°Abs as detection reagents. Since a wide variety of mAbs are available, each with their own singular IgG subclass, it is possible to mix and match various combinations of mAbs in simultaneous multiplexing experiments. While there still remain practical limitations to this approach, as reliable and specific mAbs to every target protein are not yet available in every IgG subclass, the continued pursuit of large-scale projects aimed at generating high quality mAbs, for example the NIH-funded Common Fund Protein Capture Resource (http://commonfund.nih.gov/proteincapture), the UC Davis/NIH NeuroMab Facility (http://neuromab.ucdavis.edu), and the European Union-funded Affinomics initiative (http://www.affinomics.org) will likely yield multiple specific mAbs, each of a distinct IgG subclass, against each target, facilitating their application in multiplexing experiments. Moreover, as recombinant mAbs begin to gain wider application, it becomes realistic to consider generating IgG subclass variants of any individual mAb by swapping the recombinant heavy chain constant regions that define the IgG subclass. Note that in this regard mouse mAbs offer a huge advantage for multiplex labeling over rabbit mAbs, which have only one form of IgG [7], greatly limiting their utility in multiplex labeling.

We also described an unanticipated advantage to using SCS 2°Abs, in that they yield more reliable detection of mAbs than HL preparations did in every application tested, regardless of supplier, form, conjugate and method of detection. This may seem an obvious result, in that every mAb is by definition a single IgG subclass, and one would expect higher signal and lower background when employing SCS 2°Ab preparations in which all of the 2°Ab present recognizes 1°Abs of only that subclass. In comparison to HL preparations that contain 2°Abs recognizing subclasses other than the one in current use, and as such could not contribute to specific signal and only to background.

We also found an unanticipated drawback to using HL 2°Abs, in that they exhibited a robust and reliable detection bias towards mAbs of specific IgG subclasses and against others, with a detection sensitivity of IgG2a>IgG2b>IgG1, regardless of supplier, form, level of adsorption, or conjugate. This is problematic from a number of standpoints. First and foremost, immunolabeling relies on the reliable detection of bound 1°Abs regardless of their specific characteristics, such that a “general” HL 2°Ab is not expected by the end user to prefer mAbs of certain IgG subclasses over others. Second, the detection bias is against the most common IgG subclass (IgG1) of all mAbs. As representative examples, the catalog of the UC Davis/NIH NeuroMab facility (as of 12/17/11) contains 278 mAbs, and a representative part of the commercial catalog of EMD Millipore (as of 9/2/11) includes 3631 mAbs (Ruben Flores-Saaib and Alejandra Solache, EMD Millipore, personal communication), which in both cases comprise ≈70% IgG1, ≈20% IgG2a and ≈10% IgG2b. For the most part, this reflects the representation of these subclasses in the circulating serum IgGs in immunized Balb/c mice [19], the strain most commonly used for generating mouse hybridomas. As such, it is a crucial consideration that the maximum utility of the vast majority (≈70%) of mAbs may remain unfulfilled, due to an inherent inability of the most commonly used detection reagents (*i.e.*, HL 2°Abs) to effectively detect their binding. Third, using general HL 2°Abs is especially problematic in mouse samples. Mouse samples can contain trace amounts of endogenous IgG, or have other characteristics that can lead to higher backgrounds when performing “mouse on mouse” labeling. It is generally recognized that this background is due to binding of IgG 2°Abs and not the mAbs. As expected, using a 2°Ab that sees only one IgG subclass reduces this background, and enhances the detection of the 1°Ab-specific signal, resulting in robust, specific and reliable staining of mouse samples with mAbs. Lastly, it raises the specter that validation of novel mAbs during screening, which is typically performed in the absence of any knowledge of IgG subclass, may be confounded by such a detection bias in general anti-IgG 2°Abs. Given this, it may be advantageous to use 2°Ab cocktails generated from balanced mixtures of the different SCS 2°Abs (such as employed in Figure 4A) when the IgG subclass of the mAb is not yet known. We suggest that secondary antibody suppliers make available premade cocktails balanced for secondary antibodies against each of the mouse IgG subclasses.

Most laboratories use a simple set of general HL 2°Abs to detect labeling with any mouse IgG mAb. Our results suggest that a threefold increase in their 2°Ab inventory is warranted, to include anti-IgG1, IgG2a and IgG2b SCS 2°Abs. The data shown here reveal that, like in many other situations in life and lab, the advantages of using a “one size fits all” approach, in this case using general IgG 2°Abs to detect mAbs, each of which is a single IgG subclass, are offset by the compromises often inherent in such an approach. Readily available detection reagents specifically tailored to the IgG subclass of the target mAbs offer not only greater flexibility for combining multiple mAbs in simultaneous multiplex labeling, but enhanced performance in any labeling application in which mAbs are employed.

## Materials and Methods

### 1°Abs

The generation and validation of anti-Kv1.2 mAb K14/16 (IgG2b) was described previously [20]. Mouse anti-Kv1.2 mAbs K14/39 (IgG1) and L76/36 (IgG2a) were generated against the same GST fusion protein (rat Kv1.2 amino acids 428–499) used to generate K14/16, and found to bind within the same peptide (rat Kv1.2 amino acids 463–480) as K14/16. The generation and validation of anti-PSD-95 mAb K28/43 (IgG2a) [21], anti-KChIP1 mAb K55/7 (IgG1) and anti-Kv4.2 mAb K57/1 (IgG1) [22], anti-Caspr/Paranodin mAb K65/35 (IgG1) [23], anti-Kv2.1 mAb K89/34 (IgG1) [24], anti-BK channel mAb L6/60 (IgG2a) [17] were described previously. mAbs against QKI (N147/6, IgG2b), Ankyrin-G (N106/36, IgG2a) and GFAP (N206A/8, IgG1) were obtained from the UC Davis/NIH NeuroMab Facility, which also distributes K14/16, K28/43, K55/7, K57/1, K65/35, K89/34 and L6/60. Control IgG1 (MOPC21, Cat #M9269), IgG2a (UPC10, Cat # M9144) and IgG2b (MOPC-141, Cat# M8894) were obtained from Sigma. Rabbit anti-Kv2.1 polyclonal antibody KC [25] was used as a counterstain in Figure 3.

### 2°Abs

Except where noted, all 2°Abs used in this study were obtained from Life Technologies, were raised in goats, were affinity-purified against the target immunoglobulins, and adsorbed/depleted against non-target immunoglobulins. Certain preparations were also adsorbed against target tissue. All anti-mouse 2°Abs were conjugated to Alexa Fluors®, with the exception of those used in Figure 6C which were conjugated to HRP. The Life Technologies anti-mouse generic anti-IgG 2°Ab preparations were (catalog number stated, Alexa fluor dye conjugates in parentheses): anti-mouse IgG H+L, reacting with IgG heavy chains and all immunoglobulin light chains, adsorbed against human IgG and serum: A-11001 (488), A-11005 (594); anti-mouse IgG H+L, reacting with IgG heavy chains and all immunoglobulin light chains, adsorbed against human IgG and serum, with additional adsorption against bovine, goat, rabbit, and rat IgG: A-11032 (594); F(ab′)_2_ fragment of A-11005: A-11020 (594). The Life Technologies anti-mouse IgG subclass-specific 2°Ab preparations were: anti-mouse IgG1, affinity-purified against the Fc portion of mouse IgG1 heavy chain, and adsorbed against mouse IgM, IgA, IgG2a, IgG2b and IgG3, and human sera and purified paraproteins: A-21121 (488), A-21127 (555), A-21125 (594), A-21240 (647); anti-mouse IgG2a, affinity-purified against the Fc portion of mouse IgG2a heavy chain, and adsorbed against mouse IgM, IgA, IgG1, IgG2b and IgG3, and human sera and purified paraproteins: A-21131 (488), A-21137 (555), A-21135 (594), A-21241 (647); anti-mouse IgG2b, affinity-purified against the Fc portion of mouse IgG2b heavy chain, and adsorbed against mouse IgM, IgA, IgG1, IgG2a and IgG3, and human sera and purified paraproteins: A-21140 (350), A-21141 (488), A-21147 (555), A-21145 (594), A-21242 (647).

The Rockland anti-mouse generic anti-IgG 2°Ab preparations were anti-mouse IgG H+L antibody pre-adsorbed, minimum cross-reactivity against bovine, chicken, goat, guinea pig, hamster, horse, human, rabbit, rat and sheep serum proteins, by catalog number with DyLight fluors in parentheses: 610-141-121 (488), 610-142-121 (549), 610-143-121 (647). The Jackson Immunoresearch anti-mouse generic anti-IgG 2°Ab preparations were Alexa 488 anti-mouse IgG H+L Antibody (115-545-003); and goat anti-mouse IgG H+L antibody with minimum cross-reactivity against rat, human, bovine, horse, and rabbit serum proteins (115-545-166).

For immunoblots, Kirkegaard & Perry Laboratories goat anti-mouse IgG H+L antibody, human serum adsorbed and peroxidase labeled (474–1806), and Antibodies Incorporated anti-mouse IgG H+L antibody F(ab′)_2_, affinity purified but not adsorbed (48-146-3H), were used.

The Life Technologies anti-rabbit 2°Ab preparation used here was Alexa Fluor® 488-conjugated goat anti-rabbit IgG H+L, reacting with IgG heavy chains and all immunoglobulin light chains, and adsorbed against human IgG and serum, mouse IgG and serum and bovine serum (A-11008).

### Multiple-label immunofluorescence labeling of brain sections

This study was approved by the UC Davis Institutional Animal Care and Use Committee and conforms to guidelines established by the NIH. Rats or mice were deeply anesthetized with sodium pentobarbital (Nembutal, 60 mg/kg i.p.) and perfused transcardially with phosphate buffered saline (PBS), pH 7.4, and 4% paraformaldehyde in 0.1 M phosphate buffer (pH 7.4). The brains were removed, cryoprotected for 18 hr in 10% sucrose, then 48 hr in 30% sucrose, frozen in a bed of pulverized dry ice, and then cut into 40 µm sections on a sliding freezing stage microtome. Sections were collected in 0.1 M NaPO buffer (PB) and processed immediately for immunohistochemistry. We blocked free-floating sections with 10% v/v goat serum in PB containing 0.3% v/v Triton X-100 (vehicle) and then incubated them overnight at 4°C in vehicle containing different combinations of 1°Abs (either rabbit pAb plus mAb, or mAbs of different IgG subclasses). We then incubated sections in Alexa-conjugated 2°Abs as detailed below. Images were obtained on a Zeiss Axiovert 200 microscope with Apotome, with the exception of Figure 1, which is conventional imaging. Imaging and post-imaging processing was performed in Zeiss Axiovision and Adobe Photoshop software, taking care to maintain any linear differences in signal intensities present in the original samples. All 1° Abs are used at their optimal concentrations specific to each 1° Ab preparation, determined empirically. All 2°Abs were used at a concentration of 1 µg/mL unless otherwise stated.

For Figure 1, Life Technologies 2°Abs were panel B: Alexa 647 anti-mouse IgG1 (A-21240, pseudocolored as green in panel E), panel C: Alexa 555 anti-mouse IgG2b (A-21247), and panel D: Alexa 488 anti-mouse IgG2a (A-21131, pseudocolored as blue in panel E). For Figure 2, 2°Abs in panels A, C, D were Alexa 488 anti-mouse IgG1 (A-21121), Alexa 594 anti-mouse IgG2a (A-21135), and Alexa 350 IgG2b (A-21140), and in panels B, E–H were Alexa 488 anti-mouse IgG1 (A-21121), Alexa 594 anti-mouse IgG2a (A-21137), and Alexa 647 IgG2b (A-21242). In some panels, individual colors were pseudocolored as noted in the figure legend. For Figure 3, Life Technologies 2°Abs in left panels were Alexa 594 goat anti-mouse IgG H+L (A-11005), and right panels Alexa 594 anti-mouse IgG1 (A-21125), anti-mouse IgG2b (A-21135), or anti-mouse IgG2b (A-21145); all panels had Alexa 488 goat anti-rabbit IgG (A-11008). For Figures 5 and 6, in all panels 2°Abs were Alexa 488 goat anti-mouse IgG H+L (A-11001), and Alexa 594 goat anti-mouse IgG1 (A-21125).

### Transient Transfection of COS-1 Cells

COS-1 cells (ATCC CRL-1650) were transfected with mammalian expression vectors for Kv1.2 [26], Kv2.1 [27] and PSD-95 [28], or empty expression plasmid using Lipofectamine 2000 (Life Technologies). Cells were seeded at 30% confluence (for immunoblot analysis) or 15% confluence (for immunofluorescence labeling) and transfected at time of seeding, and left for 18–24 h. The transfection medium was removed and, after the addition of fresh medium, the cells were incubated an additional 24 h.

### Immunoblot analyses

For Figures 1 and 4, transfected COS-1 cells were washed once with ice-cold DPBS (138 mM NaCl, 2.67 mM KCl, 1.47 mM KH_2_PO_4_, 8.1 mM Na2HPO_4_, 1 mM CaCl_2_ and 1 mM MgCl_2_) and extracted with 300 µL of ice-cold lysis buffer containing 1% v/v Triton X-100, 150 mM NaCl, 1 mM EDTA, 50 mM Tris-HCl (pH 7.4), 5 mM iodoacetamide, 5 mM NaF, 1 mM PMSF and a protease inhibitor cocktail for 10 min on ice [27]. The lysates were centrifuged at 12,000×g at 4°C for 10 min. The supernatants were mixed with 300 µL of 2× reducing sample buffer (RSB) and size-fractionated by 7.5% SDS–PAGE, together with crude RBM [27]. For Figure 5C, pure GST protein (GST-RAK: [20]; GST-PSD95/KAP1.13, [29]) preparations were boiled in RSB, and size-fractionated by 15% SDS–PAGE. Following SDS-PAGE, proteins were transferred to nitrocellulose membranes (Bio-Rad), which were blocked for 1 h with Blotto (4% w/v nonfat milk in Tris-buffered saline (TBS: 50 mM Tris, pH 7.5, 150 mM NaCl) plus 0.1% v/v Tween-20 followed by 1 h incubation with 1°Abs. After 3×10 min washes with Blotto, the membranes were incubated with the appropriate 2°Abs for 1 h. Fluorescent Life Technology/Molecular Probes 2°Abs were used for immunoblots in Figures 1, 4 and 5. In Figure 1A 2°Abs were anti-IgG1 Alexa 488 green (A-21121); anti-IgG2a Alexa 647 (A-21241) pseudocolored as blue, and anti-IgG2b Alexa 555 red (A-21241), each at 1.3 µg/mL. In Figure 4A were goat anti-mouse IgG H+L Alexa 488 green (A-11001), and a 1∶1∶1 cocktail of Alexa 647 anti-mouse IgG1 (A-21240), IgG2a (A-21241) and IgG2b (A-21242), each at 1.3 µg/mL. In Figure 5C 2°Abs were anti-IgG1 Alexa 555 green (A-21127), anti-IgG2a Alexa 488 (A-21131) pseudocolored as blue, and anti-IgG2b Alexa 647 red (A-21242), each at 1.3 µg/mL. HRP-conjugated 2°Abs for Figure 6C were Kirkegaard & Perry Laboratories 474–1806, and Antibodies Incorporated 48-146-3H (each at 100 ng/mL). After 3×10 min washes with TBS, the immunoblots were visualized directly (for fluorescent 2°Abs) or after visualization with Pierce ECL Western Blotting Substrate (Thermo Scientific; for HRP 2°Abs) in a FluorChem Q imager (Cell Biosciences).

Pure mAb preparations were boiled in RSB, and size-fractionated by 12% SDS–PAGE. Proteins were transferred to nitrocellulose, blocked for 1 h with Blotto followed by incubation with the appropriate 2°Abs for 1 h, and visualized as described above. Replicate gels were stained with Coomassie Brilliant Blue R-250.

### Immunofluorescence Staining of Transfected COS-1 Cells

For the immunofluorescence labeling shown in Figures 4C, 5B and 6A, COS-1 cells were used 48 h after transfection with Kv1.2/RBG4 plasmid. Medium was removed, and cells fixed in DPBS containing 4% formaldehyde plus 4% sucrose for 30 min at 4°C. After three washes with DPBS, nonspecific protein binding sites were blocked with Blotto plus 0.1% Triton X-100 (TX-100) for 1 h at room temperature, and then incubated with 1°Abs (K14/39 = IgG1, L76/36 = IgG2a, K14/16 = IgG2b) for 1 h at room temperature. After washing three times with Blotto+TX-100, cells were incubated with 2°Abs for 1 h at room temperature, washed three times with DPBS, and mounted in ProLong Gold (Life Technologies). All cells in three randomly chosen fields in three independent samples were imaged on a Zeiss M2 Axioimager microscope. Post-imaging processing was performed in Zeiss Axiovision and/or Adobe Photoshop software, taking care to maintain any linear differences in signal intensities present in the original samples. Average fluorescence intensity for each field was quantified with the Automeasure module in the Zeiss Axiovision software package. All 1° Abs were used at 5 µg/mL and 2°Abs were used at a concentration of 1 µg/mL unless otherwise stated.

For Figure 4C, 2°Abs were Life Technologies 2°Abs Alexa 594 goat anti-mouse IgG H+L A-11005 plus either Alexa 488 anti-mouse IgG1 (A-21121), anti-mouse IgG2a (A-21131), or anti-mouse IgG2b (A-21141). For Figure 6A, Life Technologies 2°Abs in left panel were Alexa 488 anti-mouse IgG1 (A-21121), anti-mouse IgG2a (A-21131), or anti-mouse IgG2b (A-21141) with either Alexa 594 goat anti-mouse IgG H+L A-11005, Alexa 594 goat anti-mouse IgG H+L highly adsorbed A-11032, or Alexa 594 goat anti-mouse IgG H+L F(ab′)_2_ A-11020. In middle panel, Life Technologies 2°Abs were Alexa 488 goat anti-mouse IgG H+L A-11001 with either Alexa 555 anti-mouse IgG1 (A-21127), anti-mouse IgG2a (A-21137), or anti-mouse IgG2b (A-21147); Alexa 594 anti-mouse IgG1 (A-21125), anti-mouse IgG2a (A-21135), or anti-mouse IgG2b (A-21145); or Alexa 647 anti-mouse IgG1 (A-21240), anti-mouse IgG2a (A-21241), or anti-mouse IgG2b (A-21242). In right panel, 2°Abs were Rockland DyLight 549 (610-142-121) or DyLight 647 (610-143-121) goat anti-mouse IgG H+L with Life Technologies Alexa 488 anti-mouse IgG1 (A-21121), anti-mouse IgG2a (A-21131), or anti-mouse IgG2b (A-21141), or Rockland DyLight 488 (610-141-121) goat anti-mouse IgG H+L with Life Technologies Alexa 594 anti-mouse IgG1 (A-21125), anti-mouse IgG2a (A-21135), or anti-mouse IgG2b (A-21145).

### FLISAs

For FLISAs in Figures 4B, 5C and 6B, microplates were coated with 50 µL of a 50 µg/mL solution of GST-RAK fusion protein [20], washed with TBS, and blocked with Blotto with 0.1% Tween-20 overnight at 4°C, and then incubated with 1°Abs (K14/39 = IgG1, L76/36 = IgG2a, K14/16 = IgG2b) diluted in Blotto with 0.1% Tween-20 for 1 h at room temperature. After washing three times with Blotto with 0.1% Tween-20, plates were incubated with Alexa 488-conjugated 2°Abs for 1 h at room temperature, washed three times with PBS, and then scanned on a Biotek FLx800 fluorescence microplate reader. For Figure 4C, Life Technologies Alexa 488 goat anti-mouse 2°Abs were used, either anti-IgG1 (A-21121), anti-IgG2a (A-21131) and anti-IgG2b (A-21141), or anti-IgG H+L (A-11001). All 1° Abs were used at 5 µg/mL and 2°Abs were used at a concentration of 1 µg/mL unless otherwise stated.
